# Immune Response in *Pneumocystis* Infections According to the Host Immune System Status

**DOI:** 10.3390/jof7080625

**Published:** 2021-07-31

**Authors:** Eléna Charpentier, Sandie Ménard, Catherine Marques, Antoine Berry, Xavier Iriart

**Affiliations:** 1 Department of Parasitology-Mycology, Toulouse University Hospital, 31059 Toulouse, France; berry.a@chu-toulouse.fr; 2 Infinity, Inserm, CNRS, University of Toulouse III, 31024 Toulouse, France; sandie.menard@inserm.fr (S.M.); catherine.marques@inserm.fr (C.M.)

**Keywords:** *Pneumocystis*, lymphocyte, immune response, immunosuppression, TCD4, TCD8, B cells, inflammation, pneumocystosis

## Abstract

The host immune response is critical in *Pneumocystis* pneumonia (PCP). Immunocompetent hosts can eliminate the fungus without symptoms, while immunodeficient hosts develop PCP with an unsuitable excessive inflammatory response leading to lung damage. From studies based on rodent models or clinical studies, this review aimed to better understand the pathophysiology of *Pneumocystis* infection by analysing the role of immune cells, mostly lymphocytes, according to the immune status of the infected host. Hence, this review first describes the immune physiological response in infected immunocompetent hosts that are able to eliminate the fungus. The objective of the second part is to identify the immune elements required for the control of the fungus, focusing on specific immune deficiencies. Finally, the third part concentrates on the effect of the different immune elements in immunocompromised subjects during PCP, to better understand which cells are detrimental, and which, on the contrary, are beneficial once the disease has started. This work highlights that the immune response associated with a favourable outcome of the infection may differ according to the immune status of the host. In the case of immunocompetency, a close communication between B cells and TCD4 within tertiary lymphocyte structures appears critical to activate M2 macrophages without much inflammation. Conversely, in the case of immunodeficiency, a pro-inflammatory response including Th1 CD4, cytotoxic CD8, NK cells, and IFNγ release seems beneficial for M1 macrophage activation, despite the impact of inflammation on lung tissue.

## 1. Introduction

*Pneumocystis jirovecii* pneumonia (PCP), otherwise known as pneumocystosis, remains nowadays one of the most frequent fungal infections in immunocompromised subjects with a worldwide prevalence of over 500,000 cases per year [1]. The number of PCP cases in HIV-infected subjects has decreased in the last decades with the introduction of triple antiretroviral therapy, but it has increased among HIV-negative patients in relation to the increasing use of immunosuppressive therapies. Despite the efficiency of molecular diagnosis and cotrimoxazole therapy, PCP mortality rate remains high, between 20% and 30% [2].

Over a hundred years after the first description of *Pneumocystis*, knowledge about the fungus has progressed considerably. This micro-organism, initially identified as a protozoan parasite and designated as *Pneumocystis carinii*, has been classified as a fungus over the years and been renamed *Pneumocystis jirovecii* for the species specifically infecting humans [3]. Nevertheless, this atypical fungus remains impossible to cultivate in vitro, slowing down progress in understanding PCP, including its pathophysiology. Indeed, the understanding of *Pneumocystis* infection pathophysiology must rely either on clinical studies or experiments on animal models.

Rodents are the main experimental models for *Pneumocystis* infection with the specific species *P. murina* for mice and *P. carinii* for rats. They have proved to be good models for *Pneumocystis* infection for their numerous similarities with the human infections. Humans and rodents notably share the dichotomous asymptomatic infection or interstitial pneumonia in immunocompetent or immunosuppressed hosts, respectively, and the type of immunosuppression at risk of PCP [4].

Both clinical studies and rodent models underline a critical role of the host immune response in the disease. Indeed, a host with a functional immune system is able to control and eliminate *Pneumocystis* without clinical signs or the need for antimicrobial treatment. However, in the case of immunosuppression, the infected host is no longer able to eliminate the fungus, which then multiplies in the alveoli. In this case, an excessive and ineffective inflammatory reaction develops in the lungs, which leads to lung damage and a progressive dyspnoea, characteristic of PCP. Hence, the immune system can be either protective, if fully functional, or can be detrimental and greatly responsible for pulmonary degradation, in the case of immunosuppression [5,6].

Numerous studies have evaluated the impact of host immune response during PCP, with a frequent focus on lymphocytes, which are the determinant immune cells in this fungal infection. Lymphocytes encompass a variety of cells with different functions, among which the main ones are CD4 T cells (TCD4), CD8 T cells (TCD8), and B lymphocytes (BL). Currently, the role and importance of the different lymphocyte populations during *Pneumocystis* infection remains unclear and controversial [6,7,8,9]. Considering the importance of the host immune response in PCP, this review aimed to better understand the role and impact of the different immune elements, mostly lymphocytes, on the elimination of *Pneumocystis* and on the lung damage classically observed in PCP.

To that end, we will first describe the immune response in the case of *Pneumocystis* infections in physiological conditions with studies on infected immunocompetent rodents. Then, the objective of the second part will be to identify the immune elements required for controlling fungus growth and avoiding PCP. It focuses on specific immune deficiencies in human diseases or in rodent experiments and their consequences on *Pneumocystis* infection. Finally, we will study the effect of different immune elements in immunocompromised subjects during PCP, to better understand which cells are detrimental, and which, on the contrary, are beneficial in the context of PCP.

## 2. In Immunocompetent Subjects, *Pneumocystis* sp. Infection Is Controlled with No Observable Pulmonary Symptoms

In the course of their lives, humans will frequently and repeatedly be in contact with *P. jirovecii.* The first contact with the fungus usually occurs during childhood as evidenced by the high seroprevalence in infants [10,11]. PCR screening studies in immunocompetent asymptomatic adults revealed around 20% carriage [12]. Infected subjects with a fully efficient immune system are able to eliminate the fungus as demonstrated in longitudinal clinical studies [12,13]. Since *Pneumocystis* infection is mainly asymptomatic in immunocompetent subjects, it is rather difficult to study the immune physiological response in humans.

Our knowledge is therefore largely based on murine models. An infected immunocompetent mouse can also eliminate the fungus without noticeable symptoms, within an average of five weeks (depending on inoculum, mouse strains, and mode of infection) [14,15,16]. Nonetheless, it should be noted that the vast majority of these rodent experiments concern primary infections without any *Pneumocystis*-specific immunity, unlike most of the *Pneumocystis* infections in humans.

### 2.1. Stimulation of Resident Cells and Chemoattraction of Leukocytes

When rodents are infected with *Pneumocystis sp* through the respiratory tract, the Antigen Presenting Cells (APC) available in the lungs can recognise several wall or membrane compounds of the fungus, such as Major Surface Glycoprotein (MSG) or beta-D glucan (BDG). MSG and BDG act as Pathogen-Associated Molecular Patterns (PAMPs) that are detected by Pattern Recognition Receptors (PRR). PRR, such as the Toll-Like Receptors (including TLR2 and TLR4) and C-type lectin receptors (particularly Dectin-1 and Mincle) are expressed on the surface of APC, myeloid cells, and alveolar epithelial cells [17,18,19,20,21]. The interactions between PAMPs and PRR triggers APC activation and the production of cytokines (including IL-8, TNFα, IFN-γ), chemokines (including MCP-1), and pro-inflammatory eicosanoids, notably via the Nuclear factor kappa B (NF-κB) pathway [22,23,24]. Several host proteins, as vitronectin and fibronectin, are able to bind to beta-glucan components of *Pneumocystis* and induce an increase of macrophage inflammatory responses [25]. Stimulated dendritic cells express CCR7 on their surface, allowing them to migrate to the ganglia in order to activate the adaptive immunity [17]. Besides the PAMPs synthetized by *Pneumocystis*, Damage-Associated Molecular Patterns (DAMPs) produced by the host cells in reaction to a stress or injury also participate to the development of the inflammation by interacting, like PAMPs, with PRR [26]. Indeed, the high-mobility group box 1 protein (HMGB1) and IL-1β are increased in the Bronchoalveolar Lavage (BAL) of infected patients [27,28,29]. The increase of HMGB1 in the lungs was found correlated with an increase of IL-8 (a potent polymorphonuclear (PMN) chemoattractant) [27].

### 2.2. White Blood Cells Influx into the Lungs

Under the action of the APC and chemokines, leukocytes infiltrate the lung tissue. There is a significant increase in the cellularity of the lung tissue and the BAL, with a peak one to two weeks after infection, mainly due to an increase of lymphocytes but also to an influx of macrophages and PMN at the site of infection. Homeostasis returns progressively with the decrease of the fungal load [14].

### 2.3. Lymphocyte Immune Response

T lymphocyte characterisation in the lungs of infected immunocompetent mice showed a rapid increase of both TCD4 and TCD8 from the first week of infection, and a decrease around the third week post-infection [14,15]. In the lymph nodes, TCD4 lymphocytes also increase significantly at the end of the first week and decrease progressively, indicating the activation of an adaptive immune response after stimulation by the APC [15]. BL and immunoglobulins G and A later increase in the lungs, but their influx coincides with the decrease in fungal load [14].

Concerning the T helper cells, both Th1 and Th2 responses are stimulated with an increase in the cytokines IFNγ, TNFα and IL-4 [15,16,30]. In some studies where the ratio of the two responses has been calculated via cytokine production, via antibody isotypes after a second challenge or via histological observations, a predominant Th2 response was reported in immunocompetent mice [14,15,31,32]. According to Shellito et al., there is a 4:1 ratio of Th2:Th1 in the lungs one week after infection of immunocompetent mice, and a 2:1 ratio at the peak of infection (3 weeks post-infection). In the lymph nodes, Th2 profile is also predominant (ratios of 10:1 and 5:1 at one and three weeks post-infection, respectively) [15]. Pro-inflammatory Th17 response is also stimulated by *Pneumocystis* sp. infection [16,33]. The anti-inflammatory balance is provided by Tregs and the cytokine IL-10 which also increase in the infected lungs of immunocompetent mice [30,33].

Some studies have observed that the stage of *Pneumocystis* sp. predominantly present in the lungs (ascus or trophic forms) could influence the type of response, probably due to a difference in APC activation. The cystic stage would induce a predominant pro-inflammatory Th17 response with an influx of TCD8, while trophic forms would conversely cause a decrease in the interaction between dendritic cells and TCD4 [34].Recently, Eddens et al. reported the formation of an Induced Bronchus-Associated Lymphoid Tissue (iBALT) during *Pneumocystis murina* infection in immunocompetent mice [35]. iBALT are temporary tertiary germinal structures that appear around the bronchus in the context of infection, for instance *Influenza*, or any other context identified as a “danger” by the organism. As a germinal centre, they mimic the structure observed in lymph nodes i.e. an important conglomerate of BL surrounded by T lymphocytes and macrophages. It appears transiently two weeks after the infection and disappears with the elimination of *Pneumocystis* from the lungs [35].

Studies in immunocompetent humans are limited because *P. jirovecii* infections are usually asymptomatic. A study performed on non-immuno-depressed patients with chronic bronchial disease colonized by *P. jirovecii*, showed an increase in the blood number of total lymphocytes and TCD4, confirming the results observed in the lungs and lymph nodes of infected immunocompetent mice [36].

### 2.4. Elimination of Pneumocystis sp. by Phagocytosis and Cytotoxic Activity

Macrophages have a major role in the elimination of *Pneumocystis* [37,38]. They can phagocyte the fungus with or without opsonisation, in the latter case involving the interaction of Dectin-1 receptors with BDG [21,37]. An interaction between the pulmonary Surfactant protein A and MSG protein would also enhance macrophages binding and phagocytosis of *Pneumocystis* in mice [39,40] but not in vitro on human macrophages according to Koziel et al. [41]. Both M1 classical profile and M2 alternative profile can eliminate *Pneumocystis.* Nevertheless, macrophages that harbour a predominant M2 profile of alternative activation, i.e. through cytokine activation of the Th2 response (IL-4 and IL-13 among others), are preferentially involved in immunocompetent mice. This M2 profile is consistent with the predominant Th2 profile reported in infected immunocompetent mice [42,43].

Immune cell-mediated cytotoxicity may also play a role in Pneumocystis elimination. NK cells and eosinophils have demonstrated a fungicidal capacity in vitro against *Pneumocystis* [44,45]. In contrast, cytotoxic TCD8 (Tc1) collected from infected mice did not show any direct cytotoxic capacity against *P. murina*. They can, however, potentialize macrophage clearing capacity when co-cultured with non-polarised macrophages [46,47] and enhance NK cytotoxic activity, probably by IFNγ production [44].

## 3. Specific Immune Deficiencies and Risk of Pneumocystosis: Identification of Required Immune Cells

A deficiency in the cellular immune response is the main factor of susceptibility to the disease [48,49]. *Pneumocystis* sp. infection induces an influx and stimulation of various types of immune cells in the lungs, each with their own functions. To help decipher the cells that are truly essential for host defence against *Pneumocystis*, we reviewed the different immune deficiencies, particularly those associated with lymphocyte lineage, and their impact on the risk of PCP development.

Indeed, whether it is an induced depletion in mice or an inherited or acquired (iatrogenic or infectious) immune deficiency in humans, the development (or not) of PCP under various immunodeficiency conditions are important clues to establish the physiological mechanisms indispensable for the defence against *Pneumocystis*; all the more so if they are targeted deficiencies. 

### 3.1. TCD4 Lymphocyte Deficiency

TCD4 lymphocytes are essential cells for clearance of *P. jirovecii* infection, as demonstrated by the high risk of developing PCP in HIV-infected patients with a TCD4 blood count lower than 200 cells/mm^3^ [50,51]. Mouse models have confirmed the necessary role of TCD4 in the fight against PCP as injections of specific anti-CD4 antibodies are sufficient to induce the development of the disease in infected mice [8,52]. Recently, the anti-CD52 monoclonal antibody (Alemtuzumab) has been reported to increase the risk of PCP [53,54]. This drug targets mostly lymphocytes (B and T cells) with a long lasting effect and especially on TCD4 [55]. TCD4 < 200 cells/mL have been reported to persist months after the completion of therapy justifying a prophylaxis anti-*Pneumocystis* for 2–6 months or until TCD4 recovers to >200 cells/mL [56].

Although the major role of TCD4 during *Pneumocystis sp*. infection is well established, the actual subpopulations of TCD4 that are required to clear the fungus are not identified. Indeed, TCD4 population contains various heterogeneous subgroups with specific functions that can even be antagonistic. The different T helper cells (Th1, Th2, Th9, Th22, Tfh) and regulatory T cells (Treg) are the real conductors of the adaptive immune response through their production of cytokines, enhancing a pro-inflammatory cellular response, a humoral response, or an anti-inflammatory response.

In *Pneumocystis* infection, classical T helper cells (Th1, Th2 and Th17) appear to have little involvement in the control of the infection in physiological conditions. Indeed, in immunocompetent mice, the inhibition of Th1, Th2, or Th17 cytokines (IFNγ and IL-12b for Th1, IL-4 for Th2 and IL-17, and IL-23 for Th17) does not induce a PCP [4,16,42,57,58]. Elsegeiny et al. also evaluated the importance of Th1 and Th2 in *Pneumocystis* control by blocking the transducer and activator of transcription proteins of Th1 (STAT4-/- mice), Th2 (STAT6-/- mice), and both Th1 and Th2 (double KO) and confirmed that they have little impact [4]. Concerning the more recently described Th9, its inhibition (IL-9-/- mice) appears to reduce *Pneumocystis* burden with an increased Th17 response [59], suggesting it is not required for *Pneumocystis* elimination.

Follicular helper TCD4 (Tfh), which are mainly involved in B cell selection and survival [60], have also been little studied so far in *Pneumocystis* infection. Yet, they might have an important role to play as mice deficient in IL-21 (which is highly secreted by Tfh) and patients with a mutation of IL-21 receptor are susceptible to developing PCP [4,61].

The contribution of Tregs to *Pneumocystis* infection is not clearer. In humans, subjects with IPEX syndrome (Immune dysregulation, Polyendocrinopathy, Enteropathy, X-linked syndrome) present a deficiency in the Treg-specific transcription factor FoxP3 and sometimes develop PCP [62]. However, rodent studies show that specific depletion of IL-10 or Tregs leads to a faster clearance of the fungus with a higher inflammation [30,63,64]. Only Rong et al. find a delayed clearance in the absence of IL-10 [33].

PD-1 and CTLA4 are immune checkpoints that provide inhibitory signal to immune cells, among which activated lymphocytes [65]. Checkpoint inhibitors, anti-Pd1 (prenbrolizumab, nivolumab) or anti-CTLA4 (ipilimumab, tremelumab), have not been directly associated with PCP but the glucocorticosteroids adjunction for the toxicity of these pro-inflammatory therapies can increase the risk of PCP [66,67].

In the context of PCP, the focus has long been on TCD4 depletion, mostly because of their remarkable correlation with PCP risk in HIV-infected patients. Nevertheless, the TCD4 threshold of 200 cells/mm^3^ does not appear to be sufficient to predict the risk of PCP in HIV-negative patients [68]. No effector T helper or Treg have yet been specifically identified as necessary to clear *Pneumocystis*. Thus, the critical role of TCD4 could be linked to the communication with other cells, notably BL, as suggested by the observations of iBALT in immunocompetent host infected with *Pneumocystis*, containing closely intertwined B and T cells [35].

### 3.2. B-Lymphocytes Deficiency

The role of BL in PCP has little been studied compared to TCD4 lymphocytes. However, there are many arguments in favour of an essential role of these cells in the physiological response against *Pneumocystis*.

Indeed, µMT-/- or Jh-/- mice deficient in mature BL are unable to eliminate *P. murina* and develop PCP [11,69,70]. In humans, new therapies used in onco-hematology targeting B cells have also been associated with a risk of PCP. A treatment with an anti-CD20 monoclonal antibody (such as Rituximab—Rituxan® or Mabthera®), with an inhibitor of Bruton tyrosine kinase (ibrutinib) or with an anti-CD19 (blinatumomab) specifically targeting BL may increase the risk of developing PCP [71,72,73]. Idelalisib, targeting the phosphoinositide-3-kinase (PI3Kδ) of B cells and used in lymphoproliferative diseases could as well increase the risk of PCP [74,75]. However, this monoclonal antibody also has an impact on T lymphocytes and dendritic cells [76].

BL deficiency implies a dysregulation of several immune mechanisms, including antibody production, but also impaired co-activation of TCD4 by BL. Both for the isotypic switch of antibodies (from IgM to IgG, IgA and IgE classes) or for the activation of TCD4 cells, contact between BL and TCD4 cells is necessary, via the major histocompatibility complex (MHC) class II and the CD40L/CD40 system [77,78].

*Pneumocystis*-specific antibodies seem to play a partial and dispensable role in the defence against the fungus. Depletion of FcyR (Receptor for the Fc region of IgG) in the mouse model increases the fungal load but *Pneumocystis* is ultimately eliminated [79]. Depletion in the complement system does not modify the fungal load or the symptomatology of the mice [79]. In addition, PCP is rare in subjects with Bruton’s disease (otherwise known as X-linked agammaglobulinemia) or with STAT3 deficiency, in which there is a very significant decrease of immunoglobulins levels [80,81,82,83].

However, the interactions between BL and TCD4 appear to be critical for the control of *Pneumocystis* sp. infection. In humans, the hereditary CD40L immune deficiency (present on TCD4) belonging to “X-linked hyper IgM syndromes” is at high risk of PCP [84,85]. Similarly, in mice, targeted depletion of MHC II or CD40 on BL induces PCP infection [79,86]. Moreover, transfer of activated TCD4 from wild type mice but not Jh-/- mice (deficient in BL) to infected RAG-/- mice allows the elimination of the fungus [87]. This confirms the necessary action of BL in order to correctly activate TCD4 during *Pneumocystis* infection.

In mice deficient for B cells functions (depleted of B-cell Activating Factor Receptor—BAFF-R-/-), Rong et al. showed that during *Pneumocystis* infection, Th1 and Th17 responses increase significantly in parallel with the development of PCP in mice. The transfer of healthy BL in these mice reduces the overexpression of Th1 cytokines while decreasing the fungal load. This would be linked to the production of anti-inflammatory IL-10 by the BL, which inhibits Th1 and Th17 responses [88].

To summarise, B cells have a critical role during PCP infection and their main implication may more concern the antigen presentation and TCD4 activation, rather than antibody production.

### 3.3. TCD8 Lymphocyte Deficiency

Unlike TCD4 and BL, isolated TCD8 deficiency is not a risk factor for PCP. Infected mice depleted of their TCD8 by injection of specific anti-CD8 antibodies can eliminate the fungus with no delay in clearance and no observable lung histological lesions [8]. This suggests that TCD8 does not play an essential role in the clearance of the fungus in the presence of all other immune cells.

### 3.4. NK Lymphocyte Deficiency

NK lymphocytes have been little considered in the context of PCP until now. However, Kelly et al. observed that mice depleted only in NK lymphocytes (yc-/-) showed a high fungal load at 4 weeks post-infection comparable to that observed in the case of TCD4 depletion [44].

In addition, Warschkau et al. found an in vitro decrease in the production of IFNγ by splenic cells in contact with *P. murina* in the absence of NK lymphocytes, which reveals a role of these cells in the activation of the adaptive immune reaction against the fungus [89].

### 3.5. Other Immune Cell Depletions

In contrast to combined immune deficiencies (CID) or severe combined immune deficiencies (SCID) affecting more or less the entire lymphocyte lineage, subjects with neutrophilic immunodeficiency syndromes (NIDs), such as chronic septic granulomatosis, are not (or only slightly) at risk of developing a PCP [90]. Using an animal model, Swain et al. found no increased susceptibility to PCP in the case of quantitative depletion of polymorphonuclear cells (PMN) in the lungs (CXCR2 KO) or qualitative depletion of reactive oxygen production by PMN (GP91phox KO, phox/INOS KO) [91].

An eosinophilia in blood or BAL is sometimes observed in PCP among HIV-infected patients or transplanted patients [92,93,94]. Specific depletion of eosinophils in mice leads to a higher fungal load 2 weeks post-infection [45]. However, although it appears that eosinophils may have fungicidal activity on *Pneumocystis*, current data does not show whether these cells are simply able to accelerate clearance of the fungus or whether their specific depletion may cause PCP [45].

The reduction of macrophages in *P. carinii* infected rats is associated with a marked increase in the fungal load, demonstrating their importance in the clearance of the fungus [38]. M1 polarisation does not seem essential in the fungus clearance as a depletion of NF-κB nuclear factor pathway in mice does not prevent the elimination of the fungus, with a compensatory higher M2 polarisation [95,96]. Similarly, patients with inherited deficiency of the protein NEMO (NF-κB Essential Modulator), which normally activates the NF-kB pathway, present little risk of PCP [97]. Moreover, inhibition of TNFα, mainly produced by M1 activated macrophages, would have a limited impact on the emergence or development of PCP in mice depleted for TNFα receptor [57]. Anti-TNFα (Infliximab, etanercept, or adalimumab) would also have a limited impact on PCP development [98,99,100], In this context, the PCP risk seems to be mostly associated with another concomitant immunosuppressant factor such as glucocorticoids or with an impact of anti-TNFα on TCD4 [101].

To conclude, TCD4 lymphocytes (without known specific polarisation) and BL are essential cells to control *Pneumocystis* infection. Moreover, the communication between these two cells seems to be a critical step for an efficient adaptive immune response. The exchanges between these two types of lymphocytes can occur among the iBALT structure, as reported by Eddens et al. [35]. TCD8 cells, on the other hand, do not seem to have a necessary role under physiological conditions. Regarding innate immunity, macrophages have a decisive role, as APC and/or as effector cells by phagocytosis. Although NK cells are poorly studied in the context of PCP, they appear to be important for the control of the infection. Those data are summarised in Table 1.

## 4. Cellular Immunity during Pneumocystosis

PCP occurs in a context of immunosuppression, i.e. in conditions where at least one of the elements essential to the control of the fungus is impaired qualitatively or quantitatively. An important challenge now would be to identify in humans or in animal models, the secondary immune responses that take place in this context of altered immunity and their beneficial effect on the clearance of the infection and/or their toxicity to lung tissue.

In contrast to the second part of this review, where specific targeted depletions were evaluated during *Pneumocystis* infection, this third part focuses on immune cells roles in condition of PCP. Thus, these mice are immunosuppressed by lymphocytes depletion, anti-CD4 antibodies, or corticosteroids. Then, the evaluation of the outcome of infected mice after the modification of one or more immune elements helps to better understand the secondary immune mechanisms that take place during PCP.

This third part of the review also includes clinical studies focusing on good or bad prognosis factors associated with this disease.

### 4.1. Cellular Lung Infiltration during Pneumocystosis

During PCP in an immunocompromised patient, *Pneumocystis* is not cleared and an impairment of lung function and lung volume capacity occurs gradually [9,106]. *P. murina* infection in TCD4-depleted mice results in a much greater leukocyte infiltration than in immunocompetent mice, with a predominant influx of TCD8 and PMN [107].

### 4.2. T Lymphocytes

Lymphocytes are essential for controlling a *Pneumocystis* infection; however, they also are responsible for lung damage during PCP. Indeed, in SCID mice (depleted in all functional B and T lymphocytes), the lung damage is lower than in mice depleted only in TCD4 [9,108]. Moreover, lymphocyte reconstitution in these SCID mice induces an Immune Reconstitution Inflammatory Syndrome (IRIS) with a high risk of mortality, which is also encountered in humans [9,108].

A reconstitution without T-lymphocytes (anti-CD3 antibody) of infected SCID mice shows a marked improvement in lung inflammation with a decrease in mortality [109].

However, fully reconstituted infected SCID mice have a significant decrease of fungal load in comparison to T cell-depleted mice [109]. Therefore, the T lymphocyte response appears to be essential for the elimination of *Pneumocystis* while at the same time being responsible for lung tissue damage during PCP.

One can postulate that some T lymphocyte populations are actually beneficial for *Pneumocystis* elimination when others are toxic, indicating the need for further studies on TCD4 and TCD8 subpopulations.

### 4.3. TCD4 Lymphocytes

TCD4 lymphocytes have a decisive role in the control of *Pneumocystis sp*: isolated reconstitution of activated TCD4 (from *Pneumocystis*-infected mice) in SCID mice is sufficient to eliminate the fungus [109,110]. Their diminution is also associated overall with a poor prognosis of the disease [111]. However, TCD4 restoration does not have only beneficial effects as their transfer alone or with the whole lymph node cells induces a strong inflammation, potentially leading to the death of infected mice [109,110]. In humans also, a rapid reconstitution of TCD4 during PCP, in patients with AIDS, can lead to a potentially lethal immune reconstitution inflammatory syndrome (IRIS) [112].

Moreover, the isolated presence of inactivated TCD4 without BL and TCD8 was also shown to be detrimental in a mouse model without clearance of the fungus [103]. If previously activated (from an interaction with other lymphocytes), TCD4 are thus sufficient to clear *Pneumocystis* but are associated with lung inflammation.

Several studies focused on the TCD4 subpopulations, and particularly the Th1/Th2 balance, in order to understand which profile is associated with a better outcome of PCP.

First, several studies revealed the value of a Th1 profile subpopulation in clearing *Pneumocystis* from the infected lungs during PCP. Indeed, the addition of IL-12 (which stimulates Th1 differentiation) in infected mice depleted in TCD4 accelerated the fungus clearance and decreased mouse mortality. IL-12 addition was associated with an increased number of macrophages, TCD8 and PMN in the lungs, and higher IFNγ production [113]. Similarly, the direct addition of IFNγ intranasally or by adenovirus helped to control the infection with *Pneumocystis* elimination in TCD4-depleted mice [7,114]. The inhibition of PD-1 immune checkpoint in mice receiving corticosteroids have been shown to promote Th1/Th17 response leading to an enhanced macrophage activity and a better fungal clearance [115]. In humans also, a high blood proportion of Th1 TCD4 has been associated with a good prognosis of PCP in patients with auto-immune and inflammatory diseases [116].

On the other hand, the pro-inflammatory Th1 response is also pointed out as a response prolonging an inadequate inflammatory state during PCP that is toxic to the lungs [109]. It is associated with the influx of cytotoxic TCD8 and neutrophils into the lungs.

For the Th2 response, although it is the predominant TCD4 profile in immunocompetent individuals infected with *Pneumocystis*, it can also be associated with damage to lung tissue and function. The deleterious aspect of this response can potentially be explained by an overproduction of mucus that can alter alveolar epithelial cells and may reduce gas exchange [32,117,118]. This response is also associated with a possible evolution towards peribronchial and perivascular fibrosis accompanied by a reconstruction of the alveolar tissue [32,117].

Thus, both unbalanced Th1 and Th2 responses could be responsible for pathological disorders. As shown by Swain et al., depletion of BL and TCD4 in infected mice results in a strong inflammatory Th1 response linked to oedema formation and albumin release in the alveoli. In contrast, BL and TCD8 depletion induces a major Th2 response which causes a significant increase in lactic dehydrogenase (LDH), which reflects an alteration of the pulmonary tissue [103].

Regarding the Treg profile, the addition of CD25+ CD4+ T cells in infected SCID mice is beneficial on inflammation but not on the fungal load, which increases with this treatment [30,119]. These results are in agreement with those of Ruan et al., who observed a decrease in pulmonary involvement, without any change in *Pneumocystis* clearance, when the anti-inflammatory IL-10 was administered intratracheally to TCD4-depleted mice [120]. However, the role of IL-10 remains controversial as, according to a publication by Qureshi et al., the addition of IL-10 in TCD4-depleted mice had no impact on fungal load or inflammation, but worsened the clinical course of infected mice [63].

In conclusion, TCD4 have a major role in *Pneumocystis* clearance during PCP but they must be activated and preferentially in the Th1 profile. This pro-inflammatory response is associated with the production of IFNγ, the influx of cytotoxic TCD8, macrophages and PMN in the lungs. The benefits of Th1 profile appear to be independent of the actual presence of TCD4 because the addition of IL-12 or IFNy in the absence of TCD4 is sufficient to improve PCP outcome [7,113,114]. An in vitro study revealed that IFNy had no direct lethal activity on *Pneumocystis* itself [121]. Thus, TCD4 action on *Pneumocystis* could rely on the production of cytokines, such as IFNγ, which would in turn stimulate other immune elements.

### 4.4. TCD8 Lymphocytes and Cytotoxic Response

In PCP, there is a great increase in the number of TCD8 in the lungs, especially if TCD4 depletion is significant [122]. Their effect on the outcome of PCP is controversial. While isolated TCD8 depletion in immunocompetent mice does not alter the fungal load (as reported above), the depletion of TCD8 in the absence of TCD4 induces an increased *Pneumocystis* load [8,9].

Conversely, increasing the number of TCD8 by administration of IL-7 in TCD4-depleted mice further reduces the fungal load [123]. TCD8 lymphocytes would therefore have a role in the elimination of *Pneumocystis* in the absence of TCD4. They would constitute a secondary route of defence against the fungus. In two clinical studies on HIV-negative patients infected with *P. jirovecii* (mostly with auto-immune diseases), an overall TCD8 decrease in peripheral blood has also been reported as a poor PCP prognostic factor [111,124].

However, TCD8 have been shown to have a deleterious activity in the lungs of infected immunocompromised mice depleted in TCD4. Indeed, in the case of double depletion of TCD4 and TCD8, the inflammation and clinical condition of the mice were less severe than in the case of isolated TCD4 depletion [95]. Furthermore, the reconstitution of SCID mice with non-specific TCD8 induces a decrease in PaO2 and a large increase in albumin leakage into lung tissue [122]. Adding activated TCD8 (taken from infected immunocompetent mice) in infected SCID mice also increases lung lesions without improving the clearance of the fungus [125].

Pulmonary infiltration of TCD8 in infected mice depleted in TCD4 is, at least in part, linked to the production of type I IFN and TNFα. Depletion of receptors for these cytokines, decreases the number of TCD8 in the lungs and improves pulmonary lesions, without effect on the fungal load [32].

From these different results, TCD8 seem to sometimes have a discordant effect on the clearance of the fungus. This might be explained by a heterogeneity of TCD8 cells population. Indeed, Mc Allister et al. showed an impact of TCD8 polarisation on PCP outcome in mice. Even if they observed a deleterious effect after total TCD8 transfer in infected SCID mice, these mice were protected from infection when they transferred Tc1-polarised (cytotoxic) TCD8 [46]. Tc1 CD8 have also been reported as good prognosis marker in patients with auto-immune disease [116].

Therefore, TCD8 seem to constitute a secondary immune response during PCP, in the case of TCD4 absence. Like TCD4 Th1 profile, Tc1 cytotoxic would be protective in the case of PCP, in relation to an increased clearance of *Pneumocystis*. Yet cytotoxic TCD8 showed no fungicidal activity against *Pneumocystis* in vitro. Nevertheless, they increased the activity of macrophages, probably with the production of IFNγ [46,47].

### 4.5. Humoral Response

Next to cytotoxic TCD8 response, *P. jirovecii* infection also induces the production of specific antibodies (anti-MSG or anti-KEX1 for example) that are measurable over time [126,127,128,129].

Contrary to studies in immunocompetent rodents, little is known about the role of BL in the case of PCP (in immunosuppression condition). However, therapy trials based on the administration of *P. murina-*specific antibodies have been carried out on immunocompromised mice with PCP. They showed a significant reduction in fungal load and a reduction in lung inflammation in treated mice [110,130]. However, the fungus was not completely eliminated and when the humoral treatment was lifted, the fungal load increased again with the reappearance of the pulmonary disease [130].

Rapaka et al. reported the value of antibodies in eliminating the fungus by opsonisation in immunocompromised mice. Indeed, the introduction of a fusion protein Dectin-1/IgG1 Fc fragment (antibody that binds to BDG) led to an improvement in the elimination of *P. murina* despite the absence of TCD4 and BL, related to an increased phagocytic capacity of alveolar macrophages [131].

Assays of immunization with *Pneumocystis* recombinant protein KEX1 on immunocompromised non-human primates induces robust and durable humoral responses [132,133]. In a pilot study, this immunization prevented the development of PCP in non-human primates infected with sHIV [133]. However, in a second evaluation with a drug-induced immunosuppression, KEX1 immunization did not prevent *Pneumocystis* infection in the primates [132].

Thus, antibodies produced by BL have shown interesting effects in PCP, both on fungal load and inflammation. However, this effect was observed transiently and was not sufficient to heal the mice from PCP. The humoral response seems to have at least an interest by increasing fungicidal phagocytic capacities of macrophages.

### 4.6. Other Immune Cells

Concerning macrophages, the increase in the number of lung macrophages and their fungicidal activity by addition of GM-CSF in the lungs of infected mice depleted in TCD4 induced a marked improvement in fungal clearance and decreased inflammation. Conversely, GM-CSF depletion in infected immunocompromised mice increased the fungal load and lung inflammation [134].

In contrast to infected immunocompetent rats harbouring predominantly M2-polarised macrophages, rats immunocompromised by corticosteroid therapy have predominantly M1-polarised macrophages [43]. Both M1 and M2 macrophages have been shown to be effective in vitro and in vivo in phagocyting and eliminating the fungus [43,102]. However, the addition of M2 macrophages in infected immunocompromised mice induces much less inflammation than the addition of M1 macrophages [43,135] while eliminating *Pneumocystis*. The macrophage orientation towards M2 response has been shown to be beneficial in infected immunocompromised mice [95,136], even in the absence of TCD4 [136]. The orientation towards M1 or M2 polarisation is influenced by Th1 (IFNγ or TNFα) or Th2 (IL4 or IL13) cytokines, respectively [42,43].

Apart from their phagocytic activity, macrophages also produce chemoattracting cytokines. In particular, they are the main producers of TNFα. When TNFα receptors (TNFR I and II) are depleted in infected TCD4-depleted mice, the production of chemokines such as MCP-1 or Regulated on Activation, Normal T Cell Expressed and Secreted (RANTES) is decreased as well as the lung infiltration of TCD8 and PMN. This reduces inflammation such as lung lesions and clinical impairment associated with the disease [137]. Hence, like TCD4 and TCD8, macrophages seem to have an important role to play in PCP as fungicidal effector cells, but with associated potential lung injury due to stimulation of tissue inflammation.

As for PMN, a high infiltration into the lungs of PCP patients, whether HIV-infected or not, has been shown to be a poor prognostic factor for the disease [138,139,140]. It is generally associated with an increase in LDH and a decrease in the number of macrophages in the lungs [93,102]. In rodents, despite a correlation between the concentration of PMN in the lungs and the severity of the disease, there is no evidence of direct PMN toxicity to lung tissue in the context of PCP. A decrease in the number of PMN in the lungs (without impact on TCD8 or macrophages) or the suppression of the production of superoxide species by PMN in TCD4-depleted mice does not improve tissue damage or respiratory function. Furthermore, these alterations in the number or functions of PMN do not modify the fungal load either, confirming the absence of cytotoxic activity of PMN on *Pneumocystis* sp. [91]. Hence, PMN concentration in the lungs seem to be an indirect marker of lung lesions without direct effect on PCP pathophysiology.

Stimulation of eosinophils by addition of IL-5 before infection in mice lacking TCD4 or completely deficient in lymphocytes leads to a better clearance of the fungus at 14 days post-infection, without any increase in the Th2 response. In the case of eosinophils depletion, the addition of IL-5 shows no more beneficial effect, confirming the implication of eosinophils [45]. Their effect on fugal load could be related to their cytotoxic activity demonstrated against *Pneumocystis* [45].

Finally, NK cells have been little studied in PCP context. Yet, they might be of interest in PCP. Their high number in the blood was reported as a good prognosis marker in HIV-negative patients [111]. NK cells have demonstrated a cytotoxic activity on *Pneumocystis* in vitro [44]. NK cells are also IFNγ-producers [141], such as Th1 TCD4 and Tc1 TCD8, and their protective effect in PCP could be related to the stimulation of TCD4, TCD8, or macrophages through cytokine production.

According to all these data (reported in Table 2), the most appropriate immune response during PCP (in conditions of immunosuppression) requires a balance between the different cell populations and between the response profiles. The impact of each immune actor on fungal clearance and on lung inflammation are often dissociated. Th1 TCD4, Tc1 TCD8 and NK cells would have a positive effect on the clearance of *Pneumocystis* but are also associated with enhanced lung inflammation. Conversely, the addition of Treg reduces lung inflammation but does not improve the clearance of the fungus. Inflammation appears to be an important process to clear *Pneumocystis* but can lead to pulmonary tissue degradation.

## 5. Conclusions

**Figure 1:**
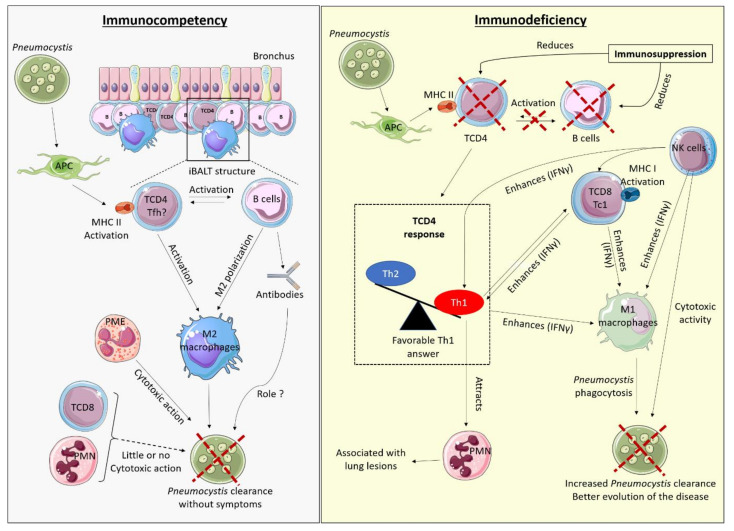
Hypothetical model of the host immune responses during *Pneumocystis* infection in the immunocompetent and immunosuppressed host. APC: Antigen Presenting Cells, iBALT: induced-Bronchus Associated Lymphoid Tissue, Tfh: T follicular helper, PMN: Polymorphonuclear neutrophils, PME: Polymorphonuclear eosinophils, MHC: Major Histocompatibility Complex. The red cross represents a reduction of the covered element. In an immunocompetent host (**left**), *Pneumocystis* is recognised by APC which activate the TCD4 response, in turn activating B cells (BL), all gathered within structures of germinal centres around the bronchus: induced Bronchus Associated Lymphoid Tissue (iBALT). TCD4 inside those iBALT structures could be T follicular helper cells (Tfh). The joint action of LBs and TCD4 activates macrophages with a preferential M2 polarisation that can then phagocytose *Pneumocystis*, with or without opsonisation (via antibodies). TCD8 and PMN have little or no action in this context, while eosinophils could intervene through their cytotoxic action on *Pneumocystis*. This immune response allows clearance of the fungus without respiratory symptoms. In an immunocompromised host (**right**), in the absence of TCD4 and/or BL populations, the immune response is not properly activated. When TCD4 response is oriented toward a predominant Th1 response, PCP outcome is favourable, with enhanced clearance of the fungus by stimulation of M1 macrophages, through cytokines production, mostly IFNγ. Similarly, TCD8 differentiated into cytotoxic Tc1 and NK cells may participate in the stimulation of macrophages towards M1 polarisation, through their IFNγ secretion. NK cells could also directly eliminate *Pneumocystis* by their cytotoxic activity on the fungus. M1 activated macrophages can in turn phagocyte *Pneumocystis*. The pro-inflammatory profile would allow better elimination of the fungus but can induce potential lesions of the lung tissue, associated with significant PMN influx.

*Pneumocystis* infection induces different immune responses depending on the immune status of the infected host and the effective cells present in the lungs. There would not be one single mechanism able to control the infection. This has already been mentioned in studies carried out in the mouse model, with the observation of a different macrophagic polarization, M2 in immunocompetent rats, and M1 in immunocompromised rats that could both eliminate the fungus [43]. A hypothetic model of immune response according to the host immune status can be proposed and is shown in Figure 1.

The immune response in the immunocompetent host seems to require very significant communication between BL and TCD4. That would be in accordance with the recently reported iBALTs observed at the peak of *Pneumocystis* infection in immunocompetent mice, which are temporary tertiary germinal structures containing BL surrounded by T lymphocytes and macrophages, usually of M2 polarisation [35].

One hypothesis could be that *Pneumocystis sp*. present in the alveolar region can induce an initial response via epithelial cells and APC, including dendritic cells. The APC would then activate the specific TCD4 by presenting them with the antigens of the fungus, via MHC II. A co-activation of BL and TCD4 may next occur in iBALT structures. TCD4 could move towards a differentiation profile, perhaps Tfh, favouring close communication with BL (via CD40) and formation of germinal centres in the presence of IL-6, TGFβ, and ICOS. TCD4 and BL would activate macrophages and direct them towards M2 polarisation, perhaps through IL-10 production by BL. Activated M2 macrophages would thus eliminate the fungus by phagocytosis, with or without opsonisation.

This hypothetical model is consistent with existing literature on PCP risk factors associated with lymphocyte populations (in mice and humans) and described in this review. Indeed, the immune response based on iBALT formation requires the communication between BL and TCD4, which has been characterised as crucial for controlling *Pneumocystis* sp. infection in both humans and mice [50,52,71,84,86]. Although iBALT formation is reduced in the absence of Th2 or Th17, this model of immune response is not directly dependent on Th1, Th2, or Th17 responses [35], consistent with the lack of PCP susceptibility of mice specifically depleted in IFNγ, IL-4, and IL-17 cytokines [16,42,105]. In contrast, regulatory TCD4 may have an important role in the differentiation of TCD4 Tfh cells that require a TGF-rich environment, consistent with observation of PCP in the case of FoxP3 deficiency or Treg depletion [30,31]. Finally, this model is linked to M2 polarisation of macrophages, as observed in immunocompetent mice [42,43]. In contrast, the implication of antibodies is not clearly determined but their role in the elimination of *Pneumocystis* could be limited, considering the low risk of PCP in patients with Bruton’s disease or hereditary Stat3 deficiency [80,83]. In this model, TCD8 would have little impact on *Pneumocystis* infection in immunocompetent hosts [8].

In immunocompromised hosts, pro-inflammatory cells (Th1 TCD4, Tc1 TCD8, and NK cells) are associated with a better fungal clearance. The role of these cells in PCP seems highly related to their cytokine production, notably IFNγ, as the addition of this cytokine in the absence of TCD4 and TCD8 is sufficient to trigger the decrease of fungus load. IFNγ, for its part, don’t seem to have any direct lethal effect on *Pneumocystis*. However, IFNγ can activate macrophages in the M1 polarisation, which are able to phagocyte and eliminate *Pneumocystis*. Th1, Tc1, and NK cells also mediate inflammation observed in the lungs, associated to a pulmonary influx of PMN. Thus, although lung lesions observed in PCP are related to inflammation, this process seems to be of great importance in fungus clearance. This model of immune response in the immunocompromised host is consistent with some human and mouse studies that also find a favourable Th1/Tc1 response [7,30,113,116]. The ability of M1 macrophages to phagocytose *Pneumocystis* has also been demonstrated in mice [43].

In conclusion, understanding the pathophysiology and the immune mechanisms of PCP requires a clear differentiation of the role of each actor of this response depending on the immune status of the host infected by *Pneumocystis*. Indeed, the role and impact of each immune cell on the outcome of *Pneumocystis* infection seem to depend on the immune status of the host: a given response may be favourable or unfavourable depending on the existence or not of an immunosuppression. It is also likely that the type of immunosuppression may induce different secondary immune responses. Understanding these different types of response will be necessary for a more individualised and optimal management of PCP patients according to the underlying immunosuppressive factors.

## Figures and Tables

**Table 1:** Fluctuation of immune cells during *Pneumocystis* infection under physiological conditions and impact of their depletion.

	Physiological Response	Specific Depletion
Lymphocytes	Increase in lymph nodes and lungs ^‡^ [14]	**SCID or RAG2 mice** ^‡^**: PCP** [9,102] **DICS hereditary deficits** ^†^**: high risk of PCP** [48,49]
TCD4 lymphocytes	Rapid Increase in lymph nodes and lungs ^‡^ [14,15]	**Anti-CD4 antibodies** ^†^: **PCP** [8,52] **TCD4 < 200/mm^3^: high risk factor for HIV-infected patients** ^†^ [50,51]
TCD8 lymphocytes	Influx in the lungs ^‡^ [14] No fungicidal activity of Tc1 CD8 ^‡^ [46,47]	Anti-CD8 antibodies: no PCP, no inflammation ^‡^ [8]
B lymphocytes	Delayed influx in the lungs ^‡^ [14] Organised in germinal centres structures around bronchus ^‡^ [35]	**µMT mice, Jk-/- mice or anti-CD20 antibodies**^‡^**: PCP** [86,103,104] **Anti-CD20 antibodies** ^‡^**: increased risk of PCP** [71,72] **CD40 deficit (*mice* and humans)** ^‡^^,^^†^**: PCP** [79,84,85,86]
Th1 TCD4 lymphocytes	Increased profile Minority compared to Th2 profile in the lungs and in the lymph nodes ^‡^ [14,15,31,32]	Isolated depletion of IFNγ: no PCP ^‡^ [57,105]
Th2 TCD4 lymphocytes	Increased profile Major profile in the lungs and in the lymph nodes ^‡^ [14,15,31,32]	IL-4 receptor depletion: no PCP ^‡^ [42]
Th17 TCD4 lymphocytes	Increased profile Minority compared to Th1 and Th2 in the lungs ^‡^ [16,33]	Anti-IL-17 antibodies: no PCP but delayed clearance ^‡^ [16,58]
Tfh TCD4 lymphocytes	Possibly present in iBALT ^‡^ [35]	**IL-21R depletion**^‡^*,***IL-21R mutation**^†^**: PCP** [4,61]
Regulatory T lymphocytes	Increased profile in the lungs ^‡^ [30,33]	Deficiency of TCD4+ CD25+ or deficiency of IL-10 ^‡^: no PCP [30,63,64] **Hereditary deficiency of FoxP3** ^†^**: PCP** [62]
Humoral response	Delayed increase in the lungs ^‡^ [14]	Fcy receptor depletion ^‡^: no PCP, maintained clearance without inflammation [79] C5 complement depletion ^‡^: no PCP [79] X-linked agammaglobulinemia (Bruton’s disease) ^†^ or STAT3 deficiency ^†^: low risk of PCP [80,81,82,83].
NK Lymphocytes	Rapid increase in the lungs ^‡^ [14] Fungicidal activity [44] and activation of adaptative response ^‡^	**NK lymphocytes deficiency**^‡^**: PCP** [44]
Macrophages	Significant influx in the lungs ^‡^ [14] major M2profile ^‡^ [42,43] effector role for *Pneumocystis* clearance by phagocytosis ^‡^ [37]	TNFα inhibitors ^†^: possible risk factor of PCP [98,99] *TNFα* receptor depletion ^‡^*:* no PCP [57]. NFkB pathway deficit ^†,‡^: low risk of PCP (humans) [95,96] no PCP (mice) [97]
Neutrophils	Increased in the lungs ^‡^ [14] No fungicidal activity demonstrated ^‡^ [91]	Hereditary deficit of neutrophils ^†^: rare cases of PCP [90] inhibition of PMN tissue infiltration or PMN inflammatory activity depletion ^‡^: no PCP [91]
Eosinophils	Increased in the lungs ^‡^ [14] Fungicidal activity ^‡^ [45]	Eosinophils depletion ^‡^: increase in fungal *load* [45]

^‡^: data in rodents; ^†^: data in humans; bold: specific depletions inducing PCP.

**Table 2:** Role of immune cells during PCP on fungal load and outcome of the disease.

Addition	Impact on the Fungal Load	Impact on Lung Inflammation and Clinical Course
T lymphocytes	Required for clearance ^‡^ [142]	Induce inflammation ^‡^ [9,142] Good prognosis marker ^†^ [111]
TCD4 lymphocytes	Required for clearance ^‡^ [109,110]	Induce inflammation ^‡^ [103,109] Good prognosis marker ^†^ [111]
TCD8 lymphocytes	Not required for clearance Reduction of the fungal load but insufficient for clearance ^‡^ [8,123]	Induce inflammation ^‡^ [95,125] Good prognosis marker ^†^ [111]
B lymphocytes	/	/
Th1 TCD4 Lymphocytes	Reduction of the fungal load ^‡^ [7,113,114]	Induce inflammation ^‡^ [109] Good prognosis marker ^†^ [116]
Th2 TCD4 Lymphocytes	/	Noxious if overproduction of mucus or evolution towards fibrosis ^‡^^,^^†^ [32,117,118]
Th17 TCD4 Lymphocytes	/	/
Regulators TCD4 lymphocytes	No modification of the fungal load ^‡^ [30,119,120]	Reduction of inflammation ^‡^ [30,119,120]
Cytotoxic Tc1 CD8 lymphocytes	Reduction of the fungal load ^‡^ [46]	Reduction of inflammation ^‡^ [46] Good prognosis marker ^†^ [116]
Humoral response	Reduction of the fungal load but insufficient for clearance ^‡^ [110,130] Antibody opsonisation with macrophages ^‡^ [131]	Reduction of inflammation ^‡^ [110,130]
NK lymphocytes	/	Good prognosis marker ^†^ [111]
Macrophages	Reduction of the fungal load ^‡^ [134] M1 and M2 polarisation are fungicide ^‡^ [43,135]	M1 polarisation induces more inflammation than M2 polarisation ^‡^ [43,135,137]
Neutrophils	No modification of the fungal load ^‡^ [91]	Bad prognosis marker if large PMN infiltration ^†^ [138,139,140] No direct toxicity demonstrated ^‡^ [91]
Eosinophils	Reduction of the fungal load ^‡^ [45]	/

^‡^: data in rodents; ^†^: data in humans.

## Data Availability

Not applicable.
